# Effectiveness of Digital Flipped Learning Evidence-Based Practice on Nurses’ Knowledge, Attitude, and Practice: A Quasi-Experimental Trial

**DOI:** 10.3390/healthcare10071257

**Published:** 2022-07-05

**Authors:** Wen-Yi Chao, Li-Chi Huang, Hung-Chang Hung, Shih-Chang Hung, Tzung-Fang Chuang, Li-Yueh Yeh, Hui-Chen Tseng

**Affiliations:** 1 Department of Public Health, China Medical University, Taichung 406040, Taiwan; wenyichao99@gmail.com; 2 Department of Nursing, Nantou Hospital, Nantou 540234, Taiwan; n10519@nant.mohw.gov.tw; 3 School of Nursing, China Medical University, Taichung 406040, Taiwan; lichi@mail.cmu.edu.tw; 4 Department of Nursing, China Medical University Hospital, Taichung 404332, Taiwan; 5 Department of Internal Medicine, Nantou Hospital, Nantou 540234, Taiwan; h550327@yahoo.com.tw (H.-C.H.); nemo.ch@msa.hinet.net (T.-F.C.); 6 Department of Emergency, Nantou Hospital, Nantou 540234, Taiwan; shihchan@gmail.com; 7 Department of Emergency Medicine, Nantou Hospital, Nantou 540234, Taiwan

**Keywords:** evidence-based practice, flipped learning, knowledge, attitude, practice, nurses, learning, education, nursing, continuing

## Abstract

Background: Evidence-based care has become critical in raising the quality of medical facilities. The implementation of evidence-based practice helps medical practitioners make better clinical decisions. Objective: The objective of this study was to investigate whether the innovative flipped teaching model could be as effective as the conventional teaching model in terms of knowledge, attitude, and practice and to confirm the continuous effect. Design: A quasi-experimental design using the flipped and conventional learning groups concurrently with repeat measurements was used. Setting: The setting was a 475-bed regional teaching hospital in Taiwan, from March to July 2020. Participants: The study included 114 licensed nurses who had worked longer than three months, with 57 participants each in two groups. Methods: The participants were assigned to two groups using a block randomization method. All participants completed questionnaires related to knowledge, attitude, and practice of EBP at four-time points: pre-test (T_0_) and immediately after intervention (T_1_), at month 1 (T_2_), and at month 3 (T_3_). Analysis of repeated generalized estimating equations was used. Results: The flipped and conventional learning groups had significant differences in knowledge, attitude, and practice at the T_0_ and T_1_ (*p* < 0.05). The flipped group was higher than the conventional group at T_3_ in the knowledge score (*p* = 0.001) and lower than the conventional group at T_2_ in the attitude score (*p* = 0.010). There were no significant differences between the two groups’ practice scores at different time points. There were no significantly different score changes for knowledge, attitude, and practice (*p* > 0.05). The interaction term only at T_3_ vs. T_0_ in the knowledge score was slightly different (*p* = 0.049) in primary outcome. Conclusion: The intervention methods of both groups were effective. Flipped learning is more flexible and has more time for discussion, which nurses favor. Under the policy promoted in the hospital, EBP combined with the nursing advancement system was standardized, and conventional learning also improved the learning effect.

## 1. Introduction

Evidence-based care has become critical in raising the quality of medical facilities. The implementation of evidence-based practice (EBP) helps medical practitioners make better clinical decisions by having them seek the best evidence from firmly grounded scientific research [1,2]. EBP training courses require both lectures and practice of the 5As (ask, acquire, appraise, apply, and audit) [3,4,5,6]. In several randomized controlled trials for nurses or nursing students, a series of evidence-based medicine (EBM) course workshops were provided, ranging from a few days to the entire semester [3,4,5,6,7]. However, workshops are time-intensive; they include lectures on basic knowledge and theories at the beginning of a course, followed by group discussions following the PICO (problem, intervention, comparison, outcome) framework, seeking evidence, reading and reviewing research, discussing research, combining evidence, summarizing research results, and applying the results to clinical patients by following the 5A steps: asking a question, accessing the information, appraising the articles found, applying the information, and auditing the impact [3,4,5,6]. Numerous studies have elaborated on the barriers to implementing EBP in the clinical environment, including participants’ learning attitudes, which are associated with their job’s characteristics, such as the work unit, work environment, the nurse-to-patient ratio, severity of diseases, shift schedules, and the support provided by their administrative department. Motivation for learning is also affected by medical practitioners’ demographic characteristics such as age [8], marital status, nurses’ career ladder [9], years of work experience, education level, English proficiency, and perceived workload [10]. The degree to which administrative departments are accommodative of adapting factors such as the learning environment [11], workforce scheduling, overtime policies [12], and incentives also have a bearing on the promotion of EBP learning because regular courses are time-consuming and laborious. Therefore, promoters of clinical EBP education need to be creative in planning and designing courses and incentives and overcome obstacles by using flipped classrooms instead of traditional courses [7,13]. In the past, some studies have also used various flipped learning models to evaluate the effect of nurses on EBP [14,15,16,17]. The measurement target includes knowledge, attitude, skills, behavior, self-efficacy, and learner’s satisfaction. There is usually a significant improvement in the evaluation score after the interventional educational strategy [14,16,17]. However, there are still shortcomings in various study designs, such as being limited to a single-group measurement, only descriptive questionnaires being used without interventional educational measures, a lack of persistent learning assessments, the participants being limited to a single work unit, or, in the self-learning link of flipped learning, a lack of online interaction with participants and the integration of the curriculum, so that the study may ignore passive participants [14,15,16,17].

## 2. Literatures Review

The flipped classroom teaching method originated from the design of two high school chemistry teachers in the United States [18]. They recorded a video of the lecture to transfer their learning from the classroom to their personal private space [18,19]. The tangible classroom of the flipped learning takes less time and has no restrictions on time and location. This flexibility is beneficial to nurses who work in shifts, cope with frantic clinical work and heavy workloads, and lack sufficient learning time [10,20]. Recently, nursing education has been offered with various instructional approaches, including blended learning, collaborative learning, and active learning, providing students with the opportunity to apply their knowledge in clinical settings [21]. With tools such as mobile learning, teaching platforms, and online resources, students can acquire EBP knowledge and practical experience through unlimited reviews and discussion [16,22]. That can help improve students’ attitudes toward learning and their competency in critical thinking, communication, and creative thinking. Moreover, tangible classes allow groups to acquire competency for handling complex problems, then mobile learning allows consolidation of the knowledge and skills acquired [23,24,25,26,27]. In evidence-based nursing education, the ability to solve problems and participate in learning is necessary.

Some studies have evaluated the five steps of EBM (5As) through the following dimensions: knowledge, attitude, skill, practice, and self-efficacy of nurses [1,28]. As conventional EBP education is often implemented as a series of workshops or problem-based learning activities [29], the barriers to its implementation and the time required should be considered. In contrast, flipped classroom-applied EBP has improved self-efficacy [16]. However, no studies have compared digital flipped learning and conventional learning in knowledge, attitude, and practice. Educators must develop an e-learning EBP course with only a limited number of tangible classes meant exclusively for the application, clarification, and discussion of high-level questions. Thus, this study aimed to investigate whether the innovative flipped teaching model could be as effective as the conventional teaching model in terms of knowledge, attitude, and practice and to confirm the continuous effect.

## 3. Methods

### 3.1. Study Design

A quasi-experimental design using two groups concurrently with repeat measurements was used. These participants were assigned to either the experimental group or the control group by using a block randomization method. For those randomized to the experimental group, a digital-team-based program using the flipped learning technique was adopted, whereas for those randomized to the control group, the conventional learning technique was used. The outcome measurements were knowledge, attitude, and practice of EBP, which were conducted before intervention (T_0_), immediately after intervention (T_1_), at the first month (T_2_), and at the third month (T_3_).

### 3.2. Participants

Participants were recruited by convenience sampling from a 475-bed regional teaching hospital in central Taiwan. The inclusion criterion was licensed nurses who had worked longer than 3 months. The sample size was estimated by using the EBP knowledge difference, the primary outcome, from a similar study in G Power (version 3.1). The mean differences between the differences of the two groups were 9.56 (SD = 0.89) and 15.25 (SD = 14.09), respectively [16]. With a mean difference of an alpha value of 0.05 between two groups, a power of 85%, and an effect size at 0.57, the desirable effect size was estimated at 114 participants. The total number of participants was 114, with 57 participants each in the experimental and control groups.

### 3.3. Intervention

#### 3.3.1. Interventions: Flipped Learning

The first part of flipped learning (FL), e-learning with different formats, was followed by group discussions using a team-based learning technique to solve clinical problems. Participants were required to join an online group on an instant messaging application to access seven lecture videos in the FL group. The e-learning materials focused on the concepts and applications of the EBP 5As and provided clinical case studies. The e-learning lectures covered the following topics: (1) the EBP process, (2) step 1: asking the clinical question, (3) step 2: searching for the best evidence, (4) step 3: critically appraising the evidence, and (5) step 4: applying to clinical patients. Available resources included empirical knowledge, and questions, which lasted for 10 min in each video. The content could be self-learned by the participants without any limitation of time or location, except that the first part had to be completed within two weeks. In the mobile application stage, the researcher raised questions about EBN periodically, brainstormed, and had open discussions among participants so that even inactive participants could learn from the messages.

**Figure 1:**
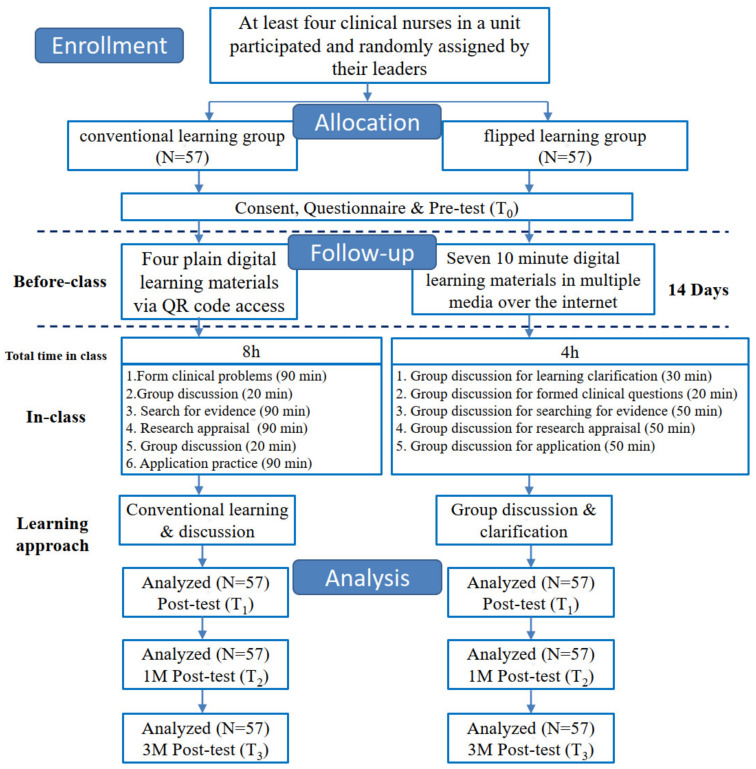
Experiment design for the conventional learning group and the flipped learning group.

After the e-learning stage, participants were required to attend tangible classes and split into six groups to discuss and provide short answers to the twelve simple questions of the quiz and instructions related to EBP. The syllabus of the tangible classroom included (1) developing the PICO based on clinical cases and presenting them in groups; (2) appraising assigned RCT and a systematic review using the tools provided in the 2012 Critical Appraisal Skills Programme [6]; (3) searching empirical papers on the Internet; and (4) planning for the clinical application of EBP. After four hours of group discussion and practice with a team-based learning technique, the results were presented orally (Figure 1).

#### 3.3.2. Interventions: Conventional Learning

In the group that applied conventional learning (CL), for the program’s first stage, participants were required to read materials. Before the classes, a QR code was provided to enable the participants to access four written handouts. The second, tangible classes focused on the concepts and applications of the EBP 5As, used a didactic instruction throughout the course, and included participants who only discussed PICO, for 20 min, and appraised papers, for another 20 min. The CL mainly consisted of instruction-based lectures for the last course. The course was completed in eight hours within a day. The study was conducted as shown in Figure 1. Participants were randomly assigned by their leaders of each unit to enter the flipped learning group or the conventional learning group and completed the pre-test after filling consent forms. Before class, participants completed digital learning. In class, they participated in the tangible class of 8 h or 4 h, respectively. After-class, three post-tests were completed immediately after intervention (T_1_), at month 1 (T_2_), and at month 3 (T_3_).

To ensure the consistency of the interventions, the same lecturer was assigned to both groups. The lecturers were core members of the Taiwanese Center for Evidence-Based Health Care who participated in the Joanna Briggs Institute Systematic Review Training Program and had ten years of experience teaching, training, and instructing EBM/EBP competition and EBP clinical practices. After the study was completed, the research team distributed the handouts and videos of educational strategies to all nurses in the hospital system.

### 3.4. Outcome Measures

The nurses’ information form included demographic variables comprising age, gender, education level, career ladder, and English proficiency. Working feature information comprised working years, unit, workload, motivation, and learning experience information. The questionnaire of knowledge, attitude, and practice was used in the evaluation of this study. The satisfaction of courses was included for understanding the participants’ responses and opinions. 

### 3.5. Instruments

The instruments to measure knowledge, attitude, and practice of EBP were adopted from Lee, Wang, and Chang’s (2011) study with a content validity of 0.86, KR-20 of internal consistency of knowledge of 0.50, and Cronbach’s α of internal consistency of attitude and practice of 0.74 and 0.93 [30]. This 30-item questionnaire included three dimensions (knowledge, attitude, and practice) and each contained 10 items to measure participants’ basic knowledge, viewpoints, and practice skills of EBP, respectively. The knowledge dimension used true or false dichotomous questions to measure, and its item ratings ranged from 0 to 10. The attitude dimension used a four-point Likert scale which ranged from 1 (strongly disagree) to 4 (strongly agree), for a total score ranging from 10 to 40. One reverse question was included, and the scores were reversed. The higher the total points, the more positive the attitude. The practice dimension used a five-point Likert scale to measure how frequently the participants implemented EBP. The item rating ranged from 1 (never) to 5 (always), for a total score ranging from 10 to 50. Higher scores indicate a better capability of EBP competence in practice 5As. The satisfaction survey included 10 question items, and the item ratings ranged from 1 (strongly disagree) to 5 (strongly agree). Higher scores indicate greater satisfaction in the learning process and course. Cronbach’s α was 0.96 [30].

### 3.6. Data Collection

The Ministry of Health and Welfare of the Republic of China Institutional review board (IRB) approval was obtained before the study was initiated (trial reference number: TPC109008). Before the study, researchers explained the education and training on EBP and obtained participants’ consent. Data were collected from March to July 2020. The same questionnaire of knowledge, attitude, and practice was administered to all participants four times in this study, at the baseline before the intervention (T_0_), immediately after intervention (T_1_), at month 1 (T_2_), and at month 3 (T_3_) (Figure 1).

### 3.7. Data Analysis

The number and percentage were used to represent the distribution of categorical variables between the CL and FL group. Mean and standard deviation (SD) were shown as the distribution of continuous variables (including age and the score of knowledge, attitude, and practice among four time points) between the two groups. The chi-square and *t*-test were used to test the difference between the two groups for categorical variables and age, respectively. We used the repeated generalized estimating equations (GEEs) model to estimate the knowledge, attitude, and practice score changes over time with the group by adjusting for career ladder and working years. An alpha level of 0.05 was designated as statistically significant.

## 4. Results

### 4.1. Comparison of Homogeneity in Demographic Variables between the Two Groups

A total of 114 nurses receiving EBP education were recruited in this study. Nurses in the CL and FL groups reported homogeneity in their demographics, working features, and learning experience. Only career ladder and working years showed significant differences (*p* < 0.05). There was no difference between the two groups for the other variables. The two groups had a similar age range: that for the CL group was 32.54 ± 8.84, and that for the FL group was 33.84 ± 7.53. Most of the participants were female nurses, and the two groups’ education levels were similarly distributed. About 65% of the participants had postgraduate degrees. In the CL group, 63.16% of the participants had a lower career ladder (N–N_1_), and 50.88% had less than five years of work experience. In the FL group, 64.91% had a higher career ladder (N_2_–N_4_), and 24.56% had less than five years of work experience (Table 1).

### 4.2. Comparison of the Knowledge, Attitude, and Practice in the Two Groups

**Figure 2:**
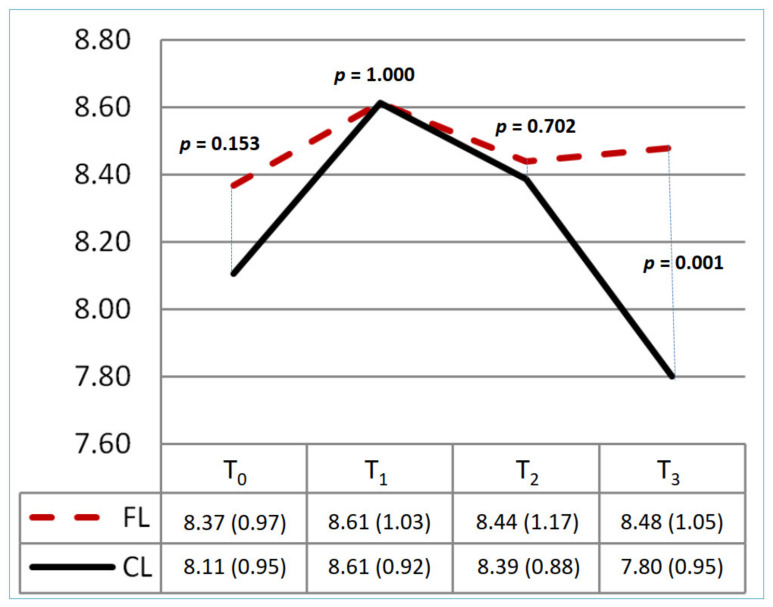
The change in knowledge score between two groups at T_0_–T_3_. CL: conventional learning; FL: flipped learning. Total range: 0–10. T_0_: baseline; T_1_: immediately; T_2_: 1st month; T_3_: 3rd month.

**Figure 3:**
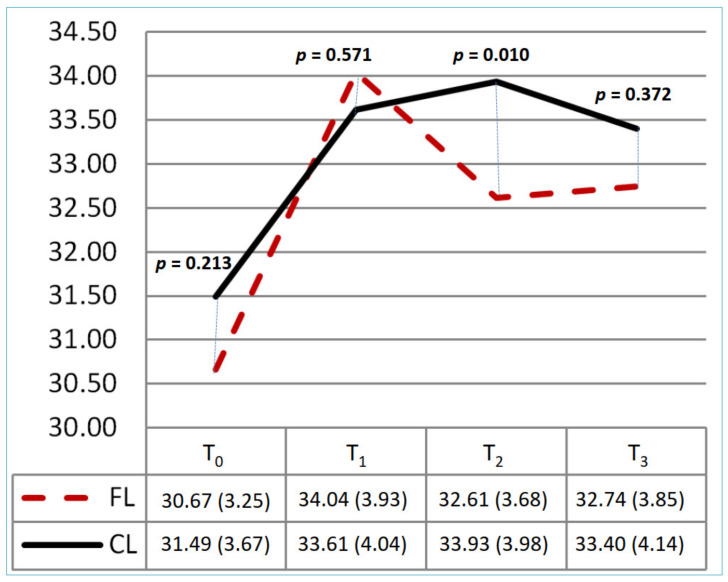
The change of attitude score between two groups at T_0_–T_3_. CL: conventional learning; FL: flipped learning. Total range: 10–40. T_0_: baseline; T_1_: immediately; T_2_: 1st month; T_3_: 3rd month.

**Figure 4:**
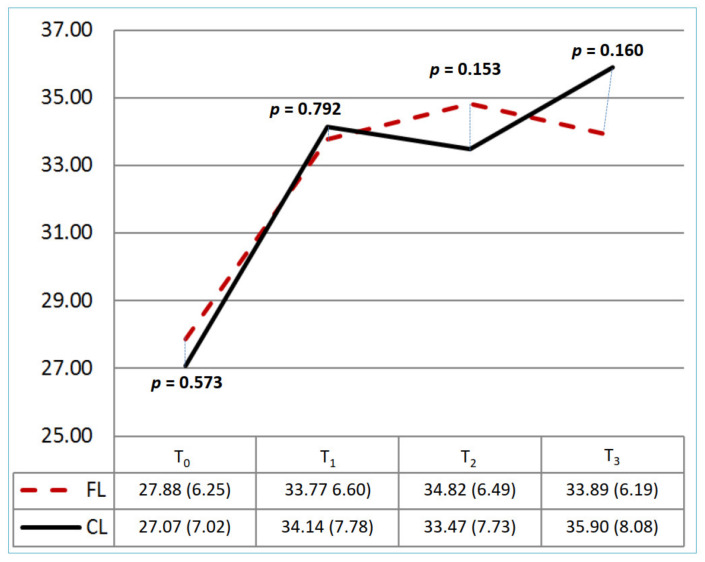
The change of practice score between groups at T_0_-T_3_. CL: conventional learning; FL: flipped learning. Total range: 10–50. T_0_: baseline; T_1_: immediately; T_2_: 1st month; T_3_: 3rd month.

Table 2 and Figure 2, Figure 3 and Figure 4 show the knowledge, attitude, and practice scores and the trend graph for the CL and FL groups at each of the four time evaluation points. There was no significant difference in the mean scores for knowledge, attitude, and practice at the baseline (*p* = 0.15, 0.21, 0.57) between the two groups. Table 2 shows the different education strategies within each group; the CL and FL groups both had significant differences in knowledge, attitude, and practice at T_0_ and T_1_ (*p* < 0.05). There was no significant difference in the two groups at T_1_ and T_2_ (*p* = 1.00, 0.702) in the mean scores of knowledge. In this trend, both groups improved at T_1_ (CL: 8.61 ± 0.92; FL: 8.61 ± 1.03); although T_2_ was gradually decreasing, it was still higher than T_0_ (CL: 8.39 ± 0.88; FL: 8.44 ± 1.17). The T_3_ score of the FL group was even higher than that of the CL group (*p* = 0.001). For the attitude score, it all improved after the intervention in both groups. The FL group had a significantly lower score than the CL group at T_2_ (*p* = 0.010), but there were no significant differences at other time points. For practice scores, it also all improved after the intervention in both groups. There were no significant differences between the two groups at different time points. 

### 4.3. Effusiveness of Intervention on Participants’ Knowledge, Attitude, and Practice of EBP

Table 3 shows the score changes between the two groups at different time points by repeated generalized estimating equations analysis. After adjusting the career ladder and working years, there were no significantly different score changes between the CL and FL groups in terms of knowledge, attitude, and practice. In the knowledge score, only T_1_ vs. T_0_ showed a significant difference (*p* < 0.0001). The attitude score significantly increased with increasing time points (2.75, 2.19, and 1.99 at T_1_, T_2_, and T_3_ vs. T_0_), and the practice score increased (6.48, 6.68, and 7.41 at T_1_, T_2_, and T_3_ vs. T_0_) (Model 1). The interaction term only at T_3_ vs. T_0_ in the knowledge score was slightly different between the two groups (*p* = 0.049) (Model 2).

### 4.4. Satisfaction of the Intervention in the Two Groups 

Table 4 shows the mean satisfaction score in the CL and FL groups pertaining to the overall curriculum design and learning process. The score for the CL group was 38.49 ± 0.99, and for the FL group was 44.24 ± 0.60. There was a significant difference in the scores between the two groups (*p* = 0.001).

## 5. Discussion

### 5.1. Knowledge of EBP

Numerous cross-sectional studies have examined knowledge, attitude, and practice concerning EBP for nurses [31,32], but few of them have introduced an educational intervention that followed knowledge, attitude, and practice [33,34]. Participants in both the FL and CL groups showed improved knowledge after intervention learning. The knowledge scores of the FL group improved significantly at the immediate post-test after the intervention learning, and the effect persisted for one month and three months. After three months, the comparison of the two groups showed that the FL score was better than that of the CL group. 

According to the flowchart of this study, the FL group was requested to proceed with seven ten-minute videos of digital learning within the two-week pre-class stage, followed by four hours in-class for 5As performance of the EBP, after which there was brainstorming and in-class deep discussion and clarification. In this study, at the beginning of the tangible class for the FL group, participants had to work in groups and provide short answers to the 12 questions on EBP. During the process, many participants used the notes they had taken when they watched the videos. As the tasks for memorizing, reciting, understanding, and absorbing are completed before the class, participants could have more time to discuss and clarify doubts in a shorter tangible class; this would also enhance their critical thinking ability [18]. Such a learning environment is conducive for nursing education, as nurses need the competency to quickly and accurately judge their patients’ assessment and decide on the necessary interventions [21]. The effectiveness of learning was enhanced by actual participation, and learning by doing strengthens memory; thus, the FL group acquired that knowledge for a more extended period [35]. The knowledge score of the FL group persisted after three months.

In the FL group, some participants may not have watched the videos. Nevertheless, the researcher raised questions about EBN periodically and conducted brainstorming and open discussions among participants so that even inactive participants could learn from the messages. Through the discussion and questions, other participants could be motivated to self-learn [24]. The teaching model designed by this study could be classified as a flipped classroom model, which is often employed in nursing education [18,36].

Given the nurses’ job characteristics, it is not easy to promote continuing clinical education. Researchers have also noted several issues with the conventional teaching methods; for example, some students may not be able to keep up with their teachers’ instructions [37], and nurses could not concentrate on the class because of shift lag [12]. However, in flipped courses, participants could watch videos without any limitation of time or location, so after the intervention learning, the effect lasted for one month and three months in this study. Teachers who have developed flipped educational plans, although increasing the time cost in the preparing stage, actually convert the time cost into low-level cognition, memory, and understanding for the students, allowing students to learn by themselves while spending time in tangible classes for the application, analysis, integration, and reflection of learning at a higher level [24]. Regardless, a benefit of flipped classrooms is the reduction in physical classroom time.

### 5.2. Attitude to EBP

Participants in both the FL and CL groups showed improved attitudes toward intervention learning. It is worth noting that the CL group significantly improved the post-test scores at one month, which were even better than those of the FL group. The possible reason was that the policy of the evidence-based reports was conducted under a request condition for an advanced clinical ladder system, which was provided by the Taiwan Nurses Association (TWNA), and it clearly defines clinical nurses’ roles and functions. Novice and beginner nurses with grading levels at N and N_1_, respectively, perform basic nursing; advanced beginners at N_2_ are competent in intensive nursing; qualified nurses at N_3_ are responsible for education and comprehensive nursing; and proficient nurses at N_4_ can conduct research and function as clinical nurse specialists (TWNA, 2018). Under the promotion policy, advanced clinical ladder N_1_ must complete the writing of EBP-related caring reports, and advanced clinical ladder N_2_ must complete an essay of EBP-related case reports. Synonyms include clinical ladder and nursing grading ladder level. In this study, 63.2% of participants were at N and N_1_ in the CL groups, during a period of positive advancement. Because of the promotion of the policy, the promotion time was consistent with the time of the second post-test in this study. Therefore, the post-test scores of the CL group at one month and three months were better than those of the FL group. As the policy was promoted in the hospital and supported by nursing executives, EBP learning and the nursing advancement system complemented each other. Nurses in medical institutions feel the importance of EBP, thus strengthening their attitude toward EBP. The attitude scale represents participants’ views, perceptions, and preconceived opinions about EBP. The higher the score, the more positive the attitude. A positive attitude can encourage students to study hard and is vital in learning [38].

### 5.3. Practice of EBP

Participants in both the FL and CL groups showed improved practice of intervention learning. Even though there was no statistical difference between the two groups at any post-test time point, performance increased over time. The improvement of practice scores in the CL group also showed the same trend as the attitude scores. The possible reason was that the policy would improve the participants’ practice of EBP in the CL group. Perhaps the policy of the advanced nursing system may have prompted EBP to be applied by the CL group, which also increased the practice score. Additionally, the research site holds an EBM competition every July, which has attracted the attention of the whole hospital to this topic. The hospital also trains two groups of new generations to participate in the competition. The competition time matched the time for the third post-test in this study. Therefore, the practice score of the CL group in the 3rd month post-test may be better than that of the FL group.

Compared to past studies, for the primary outcome, the scores of the immediate post-test of knowledge, attitude, skills, and self-efficacy were higher than those of the pre-test [14,16], while the knowledge and self-efficacy of the experimental group were significantly higher than those of the control group [16]; however, for the scores one month later, there was no difference between the two groups in knowledge and self-efficacy [16]. In terms of the learning effect between the groups, the difference of knowledge in the experimental group was higher than that of the control group [17]. To observe the effect of persistent learning, at the 6-month measurement point, the experimental group showed significantly better EBP knowledge, attitudes, behavior, and self-efficacy than the control group; at the 12-month measurement point, the improvements began to decrease [17]. Our study results were similar to those findings.

### 5.4. Satisfaction

In this study, both groups had a good response in learning satisfaction under the intervention of educational strategies. For the secondary outcome, past studies showed that the satisfaction score of participants for the flipped education teaching style provided by the educators was better than that of the control group [17]. Similar to the results of this study, the participants had good satisfaction with the learning of the EBP 5As, which was also better than that of the control group. The FL group had significantly higher scores on each item and overall satisfaction than the CL group. FL groups improve problem-solving ability by asking questions, trying to answer questions, cooperating to solve problems, and competing among themselves [24] and improve nurses’ self-efficacy and their beliefs in EBP learning [16,24], and thus improve the satisfaction of learning. Additionally, Generation Z nurses hope to use Internet resources to help them learn innovatively and independently and use digital devices to find answers to problems efficiently [39]. The key to the success of FL is the practical value and active learning preferences suitable for Generation Z [39]. The trend of satisfaction with it is also clearly seen in this study.

For future studies, we suggest that the software system of digital learning can be more efficient; educators and participants can have more interaction and practice in the system, so as to prolong the effect of learning. Moreover, the acute practice of EBP with the clinical application can be the measurement of the study result as the effective index of the continuous effect of learning, which would provide more objectively and accuracy in evaluating the effectiveness of the program.

## 6. Limitations

This study was limited due to the work of nurses because the recruited subjects matched their shift duty choice and were included in the CL group or FL group courses, so the researchers could not avoid the possibility of contamination of the research subjects. The solution was to conduct two groups of educational measures on the same day to reduce contamination. The participants’ self-administered questionnaires could lead to a reporting bias in social desirability and a lack of objective indices. We also did not investigate the actual practice experience in the study. The findings in the study cannot be generalized due to its limited sample size in one local hospital.

## 7. Conclusions

For nurses, FL is more flexible in location, tools, and time. The seven videos in the mobile application stage make self-learning livelier; there is more time for reflection, clarification, and discussion in the tangible class stage, gaining the nurses’ favor and high satisfaction; the educators can use the teaching materials in the preparatory stage repeatedly; and the teaching focus will be more on nurses’ feedback in the tangible class. However, EBP combined with the nursing advancement system was standardized. The policy was promoted in the hospital and supported by the nursing executives, which helped educators to better implement EBP education. Nurses with CL can combine the existing advanced clinical ladder system with EBP, apply EBP in clinical practice, and deepen their learning impression and practice. Therefore, in this study, both the conventional education method and the flipped education methods can improve the learning effect of nurses on EBP.

## Figures and Tables

**Table 1:** Demographic data (N = 114).

	CL (*n* = 57)	FL (*n* = 57)		
Variable	*n* (%)	*n* (%)	*t/X^2^*	*p*
Age (years), mean (SD)	32.54 (8.84)	33.84 (7.53)	−0.84	0.401
Sex			0.65	>0.999
Female	55 (96.49)	54 (94.74)		
Male	2 (3.51)	3 (5.26)		
Education level			0.04	>0.999
Diploma	20 (35.09)	19 (33.33)		
College and above	37 (64.91)	38 (66.67)		
^a^ Career ladder			8.98	0.000
N–N_1_	36 (63.16)	20 (35.09)		
N_2_–N_4_	21 (36.84)	37 (64.91)		
Working years			12.93	0.000
5 and under 5	29 (50.88)	14 (24.56)		
6–10	6 (10.53)	14 (24.56)		
11–15	7 (12.28)	17 (29.82)		
16 and above	15 (26.32)	12 (21.05)		
English proficiency			1.00	0.422
Acceptable to good and above	16 (28.07)	21 (36.84)		
Unacceptable	41 (71.93)	36 (63.16)		
Daily working hours			1.55	0.465
8	5 (8.77)	6 (10.53)		
8–9	42 (73.68)	36 (63.16)		
9–10	10 (17.54)	15 (26.32)		
Work unit			0.78	0.851
Medical, obstetrics and gynecology, pediatrics	16 (28.07)	19 (33.33)		
Surgical and operation room	8 (14.04)	9 (15.79)		
^b^ Others	19 (33.33)	15 (26.32)		
Intensive care unit	14 (24.56)	14 (24.56)		
Workload perception			0.16	0.832
Stressful and incompetent	16 (28.07)	18 (31.58)		
Competent	41 (71.93)	39 (68.42)		
Participation motivation			0.36	0.833
Interest and self−professional growth	24 (42.11)	21 (36.84)		
Unit assigned	26 (45.61)	29 (50.88)		
Work required	7 (12.28)	7 (12.28)		
Experienced literature seeking			0.14	0.700
Yes	26 (45.61)	28 (49.12)		
No	31 (54.39)	29 (50.88)		
Experienced critical appraisal			0.00	>0.999
Yes	29 (50.88)	29 (50.88)		
No	28 (49.12)	28 (49.12)		
Experienced critical appraisal in a team			0.14	0.851
Yes	24 (42.11)	26 (45.61)		
No	33 (57.89)	31 (54.39)		
Experienced learning through EBP			0.14	0.852
Yes	32 (56.14)	30 (52.63)		
No	25 (43.86)	27 (47.37)		

CL: conventional learning; FL: flipped learning; SD: standard deviation. ^a^ Advanced system of clinical professional ability of nurses in Taiwan. N1: nursing level 1, the ability to provide basic nursing care. N2: nursing level 2, the ability to provide critical and advanced nursing care. N3: nursing level 3, the ability to provide holistic care and education. N4: nursing level 4, the ability to provide specialist care and research. ^b^ Others: hemodialysis room, respiratory care ward, outpatient department, psychiatry ward.

**Table 2:** Knowledge, attitude, and practice of EBP (N = 114).

	CL Group (*n* = 57)					FL Group (*n* = 57)					Between Two Groups			
Item	T_0_	T_1_	T_2_	T_3_	*p*A	T_0_	T_1_	T_2_	T_3_	pB	*p*C	*p*D	*p*E	*p*F
	Mean (SD)				*p*-Value	Mean (SD)				*p*-Value	*p*-Value			
K	8.11 (0.95)	8.61 (0.92)	8.39 (0.88)	7.80 (0.95)	0.000 ***	8.37 (0.97)	8.61 (1.03)	8.44 (1.17)	8.48 (1.05)	0.040 *	0.153	1.000	0.702	0.001 **
A	31.49 (3.67)	33.61 (4.04)	33.93 (3.98)	33.40 (4.14)	0.000 **	30.67 (3.25)	34.04 (3.92)	32.61 (3.68)	32.74 (3.85)	0.000 ***	0.213	0.517	0.010 **	0.372
P	27.07 (7.02)	34.14 (7.79)	33.47 (7.73)	35.90 (8.08)	0.000 ***	27.88 (6.25)	33.77 (6.59)	34.82 (6.49)	33.89 (6.19)	0.000 ***	0.573	0.792	0.153	0.160

* *p* < 0.05, ** *p* < 0.01, *** *p* < 0.001. CL: conventional learning; FL: flipped learning. T_0_: baseline; T_1_: immediately post-test; T_2_: 1 month post-test; T_3_: 3 months post-test. K = knowledge; A = attitude; P = practice; SD = standard deviation. KAP score range: K = 0–10; A = 10–40; P = 10–50. *p*A: CL group pretest (T_0_: at the baseline before the intervention) vs. post-test 1 (T_1_: immediately after intervention). *p*B: FL group pre-test (T_0_: at the baseline before the intervention) vs. post-test 1 (T_1_: immediately after intervention). *p*C: the difference between differences of two groups at T_0_. *p*D: the difference between differences of two groups at T_1_. *p*E: the difference between differences between two groups at T_2_ (1st month after intervention). *p*F: the difference between differences between two groups at T_3_ (3rd month after intervention).

**Table 3:** Generalized estimating equations (GEEs) analysis of the effectiveness of interventions.

	Model 1				Model 2			
	Estimate	SE	95% CI	*p*	Estimate	SE	95% CI	*p*
Knowledge								
Group (FL vs. CL)	−0.21	0.14	−0.49, 0.07	0.13	−0.24	0.18	−0.60, 0.12	0.19
Test (Time)								
T_1_ vs. T_0_	0.38	0.08	0.22, 0.54	<0.0001	−0.02	0.26	−0.53, 0.50	0.95
T_2_ vs. T_0_	0.18	0.10	−0.02, 0.37	0.08	−0.04	0.33	−0.80, 0.52	0.68
T_3_ vs. T_0_	−0.10	0.11	−0.30, 0.11	0.36	0.52	0.30	−0.06, 1.10	0.08
Group × Test								
T_1_ vs. T_0_					0.26	0.16	−0.05, 0.58	0.10
T_2_ vs. T_0_					0.21	0.20	−0.18, 0.60	0.29
T_3_ vs. T_0_					−0.41	0.21	−0.82, −0.002	0.049
Attitude								
Group (FL vs. CL)	0.70	0.50	−0.28, 1.69	0.16	0.93	0.66	−0.37, 2.23	0.16
Test								
T_1_ vs. T_0_	2.75	0.46	1.84, 3.65	<0.0001	4.61	1.43	1.82, 7.41	0.001
T_2_ vs. T_0_	2.19	0.42	1.38, 3.01	<0.0001	1.46	1.44	−1.36, 4.27	0.31
T_3_ vs. T_0_	1.99	0.41	1.18, 2.79	<0.0001	2.20	1.43	−0.61, 5.01	0.13
Group × Test								
T_1_ vs. T_0_					−1.25	0.91	−3.04, 0.54	0.17
T_2_ vs. T_0_					0.49	0.83	−1.14, 2.12	0.56
T_3_ vs. T_0_					−0.14	0.82	−1.75, 1.47	0.86
Practice								
Group (FL vs. CL)	0.22	0.89	−1.53, 1.96	0.81	−0.58	1.27	−3.06, 1.91	0.65
Test								
T_1_ vs. T_0_	6.48	0.92	4.67, 8.29	<0.0001	4.72	2.76	−0.69, 10.1	0.09
T_2_ vs. T_0_	6.68	0.76	5.19, 8.16	<0.0001	7.49	2.01	3.55, 11.4	0.0002
T_3_ vs. T_0_	7.41	0.78	5.89, 8.93	<0.0001	3.20	2.05	−0.81, 7.21	0.01
Group × Test								
T_1_ vs. T_0_					1.18	1.85	−2.44, 4.79	0.52
T_2_ vs. T_0_					−0.54	1.52	−3.52, 2.43	0.72
T_3_ vs. T_0_					2.80	1.52	−0.18, 5.79	0.07

Model 1: after adjusted career ladder and work years. Model 2: after adjusted interaction term, career ladder, and work years. SE: standard error. CL: conventional learning; FL: flipped learning. T_0_: baseline; T_1_: immediately; T_2_: 1st month; T_3_: 3rd month.

**Table 4:** The satisfaction with the intervention in the two groups (N = 114).

Item	CL (*n = 57*)	FL (*n = 57*)	*p*
	Mean (SD)		
1. I could understand EBP through the course content.	3.75 (1.15)	4.46 (0.66)	<0.000 ***
2. I learned how to ask a clinical question.	3.82 (1.04)	4.42 (0.53)	<0.000 ***
3. The course strengthens my ability to search for the empirical literature.	3.82 (1.02)	4.46 (0.50)	<0.000 ***
4. The course improved my ability to critique the literature.	3.84 (1.01)	4.44 (0.50)	<0.000 ***
5. I could apply EBP in clinical care.	3.84 (1.01)	4.39 (0.56)	0.001 **
6. The course enhanced my confidence in the instructor in the clinical application of evidence-based nursing.	3.84 (1.01)	4.42 (0.57)	<0.000 ***
7. The case discussion helped me understand EBP.	3.88 (1.05)	4.44 (0.50)	<0.000 ***
8. Practical experience improved my ability to search for empirical literature.	3.96 (0.96)	4.44 (0.50)	0.001 **
9. Group discussions enhanced my ability to critique the literature.	3.86 (0.97)	4.40 (0.49)	<0.000 ***
10. The workshop helped me complete the EBP 5As and apply them to clinical care.	3.88 (0.98)	4.35 (0.52)	0.002 *
Total score	38.49 (0.99)	44.24 (0.60)	0.001 **

* *p* < 0.05, ** *p* < 0.01, *** *p* < 0.001. CL: conventional learning; FL: flipped learning. SD: standard deviation. Score range: 1–5, total range: 10–50. EBP: evidence-based practice.
